# Genomic analysis of xCT-mediated regulatory network: identification of novel targets against AIDS-associated lymphoma

**DOI:** 10.18632/oncotarget.3710

**Published:** 2015-03-30

**Authors:** Lu Dai, Yueyu Cao, Yihan Chen, Johnan A.R. Kaleeba, Jovanny Zabaleta, Zhiqiang Qin

**Affiliations:** ^1^ Research Center for Translational Medicine and Key Laboratory of Arrhythmias of the Ministry of Education of China, East Hospital, Tongji University School of Medicine, Shanghai, China; ^2^ Department of Microbiology/Immunology/Parasitology, Louisiana State University Health Sciences Center, Louisiana Cancer Research Center, New Orleans, LA, USA; ^3^ Department of Medicine, Louisiana State University Health Sciences Center, Louisiana Cancer Research Center, New Orleans, LA, USA; ^4^ Department of Pediatrics, Louisiana State University Health Sciences Center, Louisiana Cancer Research Center, New Orleans, LA, USA; ^5^ Department of Microbiology and Immunology, Uniformed Services University of the Health Sciences, Bethesda, MD, USA

**Keywords:** KSHV, herpesvirus, xCT, lymphoma, microarray

## Abstract

Kaposi's sarcoma-associated herpesvirus (KSHV) is the etiological agent of primary effusion lymphoma (PEL), a rapidly progressing malignancy mostly arising in HIV-infected patients. Even under conventional chemotherapy, PEL continues to portend nearly 100% mortality within several months, which urgently requires novel therapeutic strategies. We have previously demonstrated that targeting xCT, an amino acid transporter for cystine/glutamate exchange, induces significant PEL cell apoptosis through regulation of multiple host and viral factors. More importantly, one of xCT selective inhibitors, Sulfasalazine (SASP), effectively prevents PEL tumor progression in an immune-deficient xenograft model. In the current study, we use Illumina microarray to explore the profile of genes altered by SASP treatment within 3 KSHV^+^ PEL cell-lines, and discover that many genes involved in oxidative stress/antioxidant defense system, apoptosis/anti-apoptosis/cell death, and cellular response to unfolded proteins/topologically incorrect proteins are potentially regulated by xCT. We further validate 2 downstream candidates, *OSGIN1* (oxidative stress-induced growth inhibitor 1) and *XRCC5* (X-ray repair cross-complementing protein 5), and evaluate their functional relationship with PEL cell survival/proliferation and chemoresistance, respectively. Together, our data indicate that targeting these novel xCT-regulated downstream genes may represent a promising new therapeutic strategy against PEL and/or other AIDS-related lymphoma.

## INTRODUCTION

The oncogenic Kaposi's sarcoma-associated herpesvirus (KSHV, also known as Human herpesvirus 8) is a principal causative agent of several human cancers including primary effusion lymphoma (PEL), which arises preponderantly in immunocompromised individuals, in particular acquired immune deficiency syndrome (AIDS) patients [1]. PEL usually comprises transformed B cells harboring KSHV episomes and presents as pleural, peritoneal and pericardial neoplastic effusions [2]. PEL is a rapidly progressing malignancy with a median survival time of approximately 6 months even under conventional chemotherapy [3]. Furthermore, the myelosuppressive effects of systemic cytotoxic chemotherapy synergize with those caused by antiretroviral therapy or immune suppression [2-4], which supports the need to identify novel targets that could guide development of more effective therapeutic strategies. Recently, we found that the amino acid transporter, xCT, is highly expressed on the surface of patient-derived KSHV^+^ PEL cells, and targeting xCT by pharmacological inhibition or RNAi silencing induces significant PEL cell apoptosis [5]. We further demonstrated that the underlying mechanisms for this effect include regulation of both host and viral factors: (i) reduction of intracellular glutathione (GSH) synthesis and increased reactive oxygen species (ROS) production, (ii) repression of cell-proliferation-related signaling, in particular Akt pathway, and (iii) induction of viral lytic gene expression [5]. More importantly, we demonstrated that an xCT selective inhibitor, Sulfasalazine (SASP), effectively prevents PEL tumor progression in an immune-deficient xenograft model [5], which suggests that xCT may represent a promising target for AIDS-related lymphomas.

The expression of xCT on the cell membrane is essential for the uptake of cystine required for synthesis of intracellular glutathione (GSH), an anti-oxidant that plays an important role in maintaining the intracellular redox balance [6, 7]. Therefore, xCT is highly expressed by a variety of malignant tumors because the uptake of cystine/cysteine from the microenvironment is crucial for cancer cell growth and viability [8-11]. Interestingly, xCT has also been identified as a fusion-entry receptor for KSHV [12, 13], which is upregulated within more advanced Kaposi's sarcoma (KS, another KSHV-related malignancy [14]) lesions containing a greater number of KSHV-infected cells [15]. We also recently reported that xCT is able to activate intracellular signaling pathways (in particular MAPK), pro-migratory cytokine release, and KSHV-infected endothelial cell invasion through induction of the 14-3-3β protein [16]. Moreover, xCT is functionally involved in the pathogenesis of other viruses and bacteria as well [17-20]. However, the mechanisms of xCT-mediated regulation of KSHV pathogenesis and tumorigenesis, including the xCT regulatory network in AIDS-related lymphomas such as KSHV^+^ PEL remain unknown. Clearly, innovative insights from this information would facilitate the identification of potential “drug target” candidates for development of new therapeutic strategies. Therefore, in the current study we used Illumina human microarray analysis to interrogate changes in the transcriptional profiles of genes in 3 KSHV^+^ PEL cell-lines treated with the xCT selective inhibitor, SASP, which led to identification of a number of novel xCT-regulated downstream genes important to PEL survival or chemoresistance.

## RESULTS

### Microarray analysis of xCT-regulated network within KSHV^+^ PEL cells

**Figure 1:**
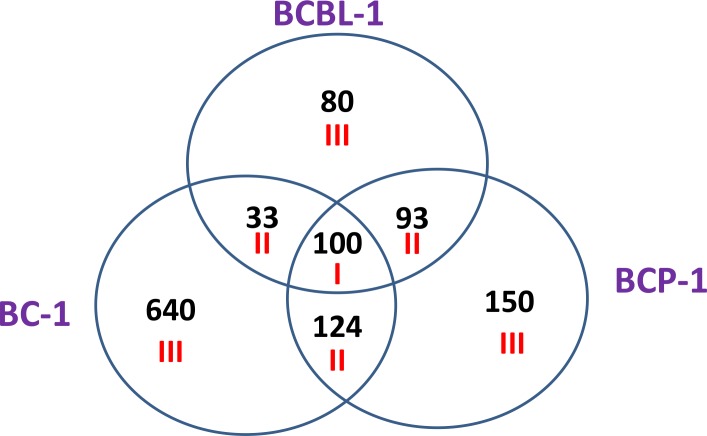
Intersection analysis of gene profile altered within SASP-treated PEL cell-lines The HumanHT-12 v4 Expression BeadChip (Illumina) was used to detect genomic gene profile altered within 3 SASP-treated PEL cell-lines (BCBL-1, BC-1 and BCP-1) when compared with vehicle-treated controls. Intersection analysis of significantly altered genes (up/down≥2 folds and *p* < 0.05) was performed using the Illumina GenomeStudio Software. Set I: Common genes altered in all the 3 cell-lines; Set II: Similar genes altered in every 2 cell-lines; Set III: Unique genes altered in each cell-line.

We first used the HumanHT-12 v4 Expression BeadChip (Illumina) which contains more than 47,000 probes derived from the NCBI RefSeq Release 38 and other sources to study the gene profile altered between vehicle- or SASP-treated 3 KSHV^+^ PEL cell-lines (BCP-1, BC-1 and BCBL-1). Intersection analysis indicated that there were totally 100 common genes significantly altered within all the 3 SASP-treated cell-lines (up/down≥2 folds and *p* < 0.05); 33 similar genes altered between BCBL-1 and BC-1, 93 similar between BCBL-1 and BCP-1, and 124 similar between BC-1 and BCP-1; 80 genes altered were unique to BCBL-1, 150 unique to BCP-1 and 640 unique to BC-1 (Figure 1). Notably, BC-1 cells, which are also EBV^+^, had a much higher number of uniquely altered genes than BCBL-1 and BCP-1. Within the common gene set, the top 20 upregulated or downregulated candidate genes in SASP-treated BCP-1, BC-1 and BCBL-1 cell-lines are listed in Table 1 and Table 2, respectively, including gene description and the altered level of transcription in these cell-lines. Interestingly, we found that the functional role of most genes in PEL pathogenesis have never been reported, although some of them have been implicated in other types of malignancies. For example, *SRXN1* (Sulfiredoxin-1), which is upregulated in all three SASP-treated PEL cell lines (Table 1) is involved in proliferation inhibition of acute myeloid leukemia mediated by Maesopsin 4-O-beta-D-glucoside, a natural compound isolated from the leaves of Artocarpus tonkinensis) [21]. Another study reported that activation of *PFKP* (6-phosphofructokinase type C), which is downregulated in SASP-treated PEL cells (Table 2) is closely associated with breast cancer cell proliferation [22]. The opposite effects of SASP on *SRXN1* and *PFKP* transcription underscores the putative benefits of this drug in clinical management of PEL as well. We next performed enrichment analysis of these common, similar and unique sets of genes using the Pathway map, Gene Ontology (GO) Processes and Process Networks modules from Metacore Software (Thompson Reuters) [23]. Our analysis shows that several major cellular functions were affected within SASP-treated PEL cells, including oxidative stress/antioxidant defense system, apoptosis/anti-apoptosis/cell death, and cellular response to unfolded proteins/topologically incorrect proteins, which is consistent with the SASP-induced apoptosis phenotype that we recently observed in KSHV^+^ PEL cell-lines [5]. The top 2 scored pathway maps and protein networks based on the enrichment analysis of “common” gene set were listed in Figures 3 and S1, respectively.

**Table 1:** The top 20 candidate genes upregulated in KSHV+ PEL cells treated by SASP

Gene symbol	Description	Fold change		
		BCP-1	BC-1	BCBL-1
OSGIN1	Oxidative stress-induced growth inhibitor 1	14.73	5.85	7.87
HSPA6	Heat shock 70 kDa protein 6	4.36	14.97	4.75
MSC	Musculin	7.51	8.53	5.46
DHRS2	Dehydrogenase/reductase SDR family member 2, mitochondrial	10.64	5.75	4.36
PPP1R15A	Protein phosphatase 1 regulatory subunit 15A	6.77	6.14	6.45
HSPA1A	Heat shock 70 kDa protein 1	7.05	5.57	5.24
SRXN1	Sulfiredoxin-1	7.53	3.03	5.85
GCLM	Glutamate--cysteine ligase regulatory subunit	6.24	4.12	5.25
SQSTM1	Sequestosome-1	7.46	2.24	5.3
RN7SK	RNA, 7SK small nuclear transcript	4.98	4.62	4.81
HSPA7	Putative heat shock 70 kDa protein 7	3.04	7.69	3.44
DNAJB1	DnaJ homolog subfamily B member 1	5.81	4.67	3.48
DNAJC3	DnaJ homolog subfamily C member 3	3.87	7.39	2.46
LILRB3	Leukocyte immunoglobulin-like receptor subfamily B member 3	4.71	3.4	4.59
RNF141	RING finger protein 141	5.23	3.3	3.86
C6orf52	Putative uncharacterized protein C6orf52	2.81	5.81	3.17
CCL3L3	C-C motif chemokine 3-like 1	4.66	4.11	2.43
ZCWPW1	Zinc finger CW-type PWWP domain protein 1	3	5.69	2.42
SLC3A2	4F2 cell-surface antigen heavy chain	4.16	4.63	2.28
DDIT3	DNA damage-inducible transcript 3 protein	3.51	2.97	4.33

**Table 2:** The top 20 candidate genes downregulated in KSHV+ PEL cells treated by SASP

Gene symbol	Description	Fold change		
		BCP-1	BC-1	BCBL-1
ASZ1	Ankyrin repeat, SAM and basic leucine zipper domain-containing protein 1	0.31	0.19	0.32
ARL4C	ADP-ribosylation factor-like protein 4C	0.25	0.33	0.25
NREP	Neuronal regeneration-related protein	0.28	0.26	0.32
SOCS2	Suppressor of cytokine signaling 2	0.28	0.23	0.39
FAM117B	Protein FAM117B	0.33	0.23	0.4
LGALS13	Galactoside-binding soluble lectin 13	0.32	0.22	0.44
PPIA	Peptidyl-prolyl cis-trans isomerase A	0.3	0.26	0.43
BTF3L4	Transcription factor BTF3 homolog 4	0.36	0.22	0.41
XRCC5	X-ray repair cross-complementing protein 5	0.41	0.11	0.47
E2F5	Transcription factor E2F5	0.35	0.31	0.35
ENPP2	Ectonucleotide pyrophosphatase/phosphodiesterase family member 2	0.32	0.28	0.41
PFKP	6-phosphofructokinase type C	0.35	0.29	0.38
RBM17	Splicing factor 45	0.4	0.21	0.42
EPB41L3	Band 4.1-like protein 3	0.36	0.3	0.38
ATP11B	Probable phospholipid-transporting ATPase IF	0.3	0.35	0.4
SERBP1	Plasminogen activator inhibitor 1 RNA-binding protein	0.42	0.15	0.48
PM20D2	Peptidase M20 domain-containing protein 2	0.47	0.21	0.37
MYLIP	E3 ubiquitin-protein ligase MYLIP	0.21	0.39	0.47
CBR4	Carbonyl reductase family member 4	0.38	0.22	0.47
TMPRSS3	Transmembrane protease serine 3	0.3	0.32	0.46

**Figure 2:**
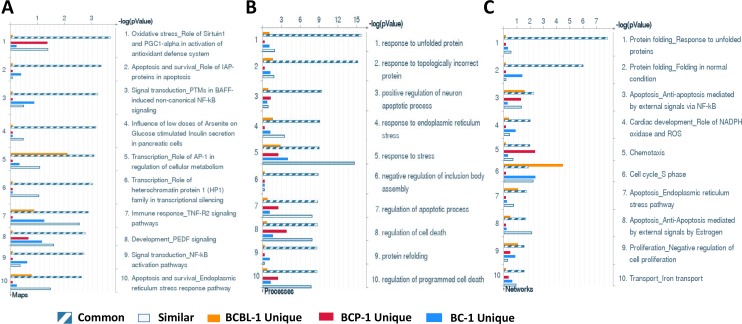
Enrichment analysis of gene profile significantly altered by inhibition of xCT within KSHV-infected PEL cell-lines The enrichment analysis of gene profile (common, similar and unique profile as indicated) significantly altered by inhibition of xCT was performed using the Metacore Software (Thompson Reuters) Modules: Pathway Maps (**A**), Gene Ontology Processes (**B**) and Process Networks (**C**).

**Figure 3:**
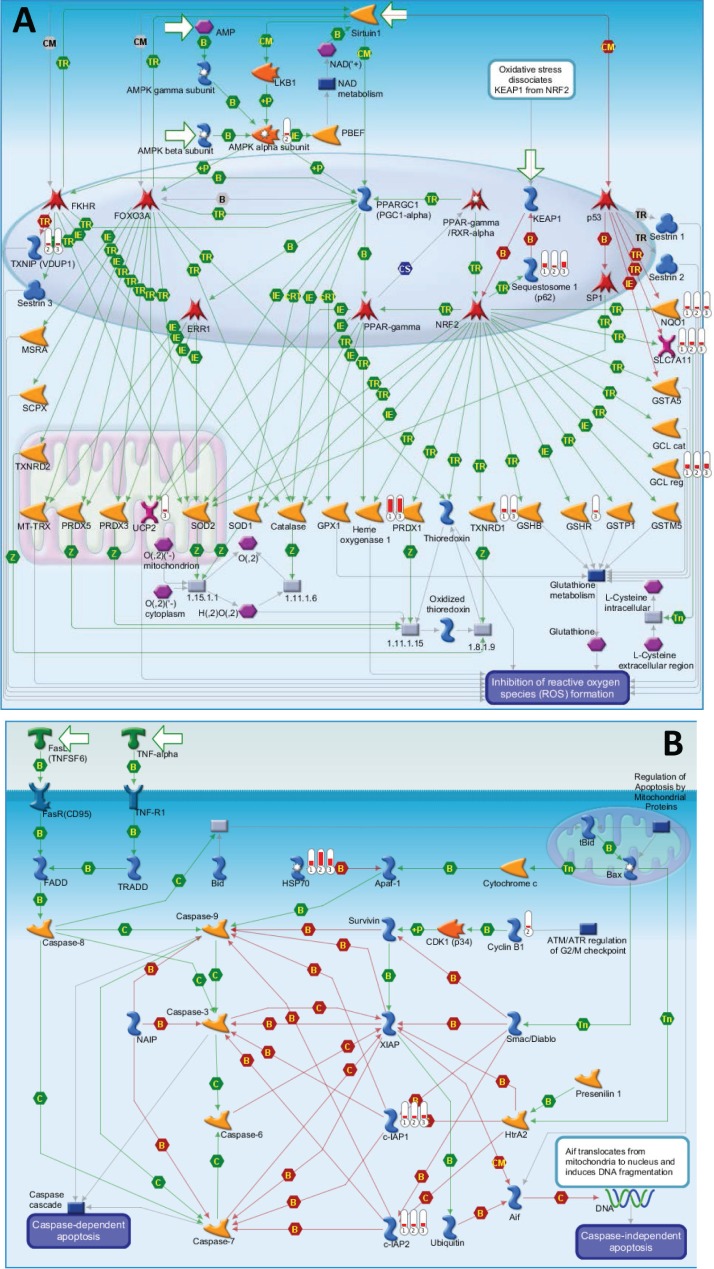
The top 2 scored maps (maps with the lowest p-value) based on the enrichment distribution sorted by ‘common’ gene set (**A**) Oxidative stress: Role of Sirtuin1 and PGC1 alpha in activation of antioxidant defense system. (**B**) Apoptosis and survival: Role of IAP proteins in apoptosis. Experimental data from all files is linked to and visualized on the maps as thermometer like figures. Up-ward thermometers have red color and indicate upregulated signals and down ward (blue) ones indicate downregulated expression levels of the genes. Data was produced by the Metacore Software (Thompson Reuters).

### Experimental validation of microarray results with selected downstream candidates

**Figure 4:**
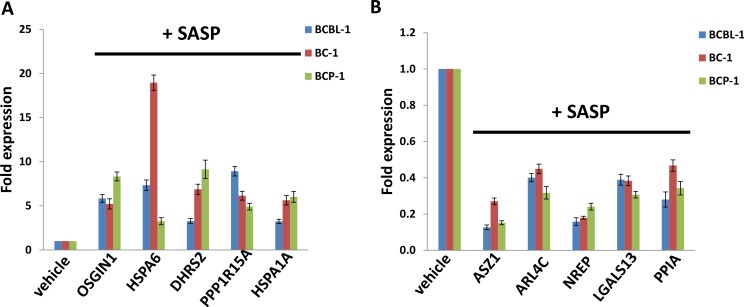
Validation of microarray results by qRT-PCR for selected candidate genes The transcriptional levels of selected 5 candidate genes upregulated (**A**) or downregulated (**B**) as shown in microarray data were validated by using qRT-PCR, respectively. Error bars represent the S.E.M. for 3 independent experiments.

We next selected 5 genes from the top 20 upregulated or downregulated candidate list (Tables 1 and 2) for validation of their transcriptional change by qRT-PCR. Our results indicated that all the 5 genes (*OSGIN1*, *HSPA6*, *DHRS2*, *PPP1R15A* and *HSPA1A*) were significantly upregulated in SASP-treated PEL cells when compared with vehicle-treated cells; while another 5 genes (*ASZ1*, *ARL4C*, *NREP*, *LGALS13*, *PPIA*) were all significantly downregulated in SASP-treated PEL cells (Figure 4). Moreover, the altered transcriptional levels of these genes in all the 3 KSHV^+^ PEL cell-lines (BCBL-1, BC-1 and BCP-1) were comparable to those found in microarray data, demonstrating the credibility of our microarray analysis.

### The role of OSGIN1 in SASP-induced KSHV^+^ PEL cell apoptosis

**Figure 5:**
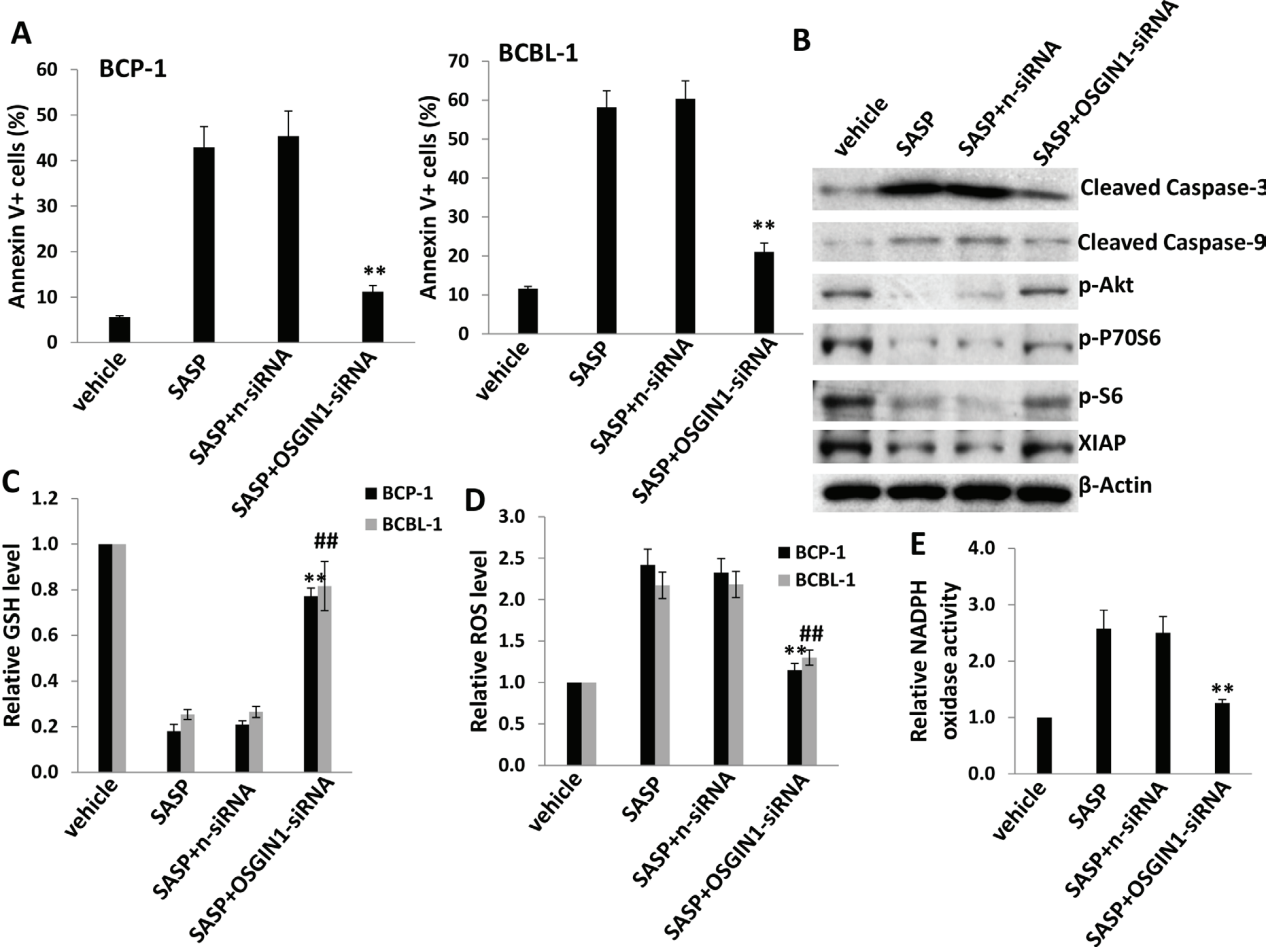
xCT inhibition induces KSHV-infected PEL cell apoptosis potentially through upregulation of OSGIN1 (**A**) BCP-1 and BCBL-1 were transfected with either negative control siRNA (n-siRNA) or *OSGIN1*-siRNA for 48 h, then incubated with 0.5mM of SASP for additional 24 h and cell apoptosis was assessed using Annexin V-PI staining and flow cytometry analysis. (**B**) Protein expression in BCBL-1 was measured by immuoblots. (**C**-**D**) The levels of intracellular GSH and ROS were quantified as described in Methods. (**E**) The activities of NADPH oxidases in BCBL-1 were measured as described in Methods. Error bars represent the S.E.M. for 3 independent experiments. **/## = *p* < 0.01 (*vs* SASP+n-siRNA group).

We next selected *OSGIN1* (Oxidative stress-induced growth inhibitor 1), one of the highly upregulated genes in SASP-treated KSHV^+^ PEL cells from microarray data, to determine its role in SASP-induced cell apoptosis. The *OSGIN1* gene (also named as *OKL38*) was first identified in breast epithelial cells as increasingly expressed during pregnancy and lactation [24]. Low-level expression of this gene has been reported in breast cancer cell lines, while its overexpression in MCF-7 breast cancer cells leads to a reduction in proliferation as well as tumor formation in nude mice [24]. As a tumor suppressor, downregulation of OSGIN1 was also found to be closely associated with progression of other malignancies such as hepatocellular carcinoma and kidney cancer [25-27]. Here, we found that silencing of *OSGIN1* by RNAi significantly reduced cell apoptosis induced by SASP (0.5 mM) in BCP-1 and BCBL-1 cells (Figures 5A and S2). Western blot analysis also indicated that silencing of OSGIN1 by RNAi in SASP-treated BCP-1 and BCBL-1 greatly reduced cleaved Caspase 3 and 9, while partially rescuing the phosphorylation of Akt, downstream P70S6, S6 and the expression of X-linked inhibitor of apoptosis protein (XIAP) [28], a physiologic substrate of Akt that is stabilized to inhibit programmed cell death (Figure 5B). We have previously shown that SASP-induced PEL apoptosis may also be orchestrated via reduction of intracellular GSH synthesis and increased ROS production [5]. Here we found that silencing of *OSGIN1* significantly restored intracellular GSH synthesis and reduced ROS production from SASP-treated cells (Figure 5C-5D). Biochemical assays further confirmed that silencing of *OSGIN1* caused a reduction in the activity of NADPH oxidase (Figure 5E), the major source of ROS production [29, 30]. We also found that silencing of xCT by RNAi upregulated *OSGIN1* transcripts in BCBL-1 cells, indicating that this gene is indeed a downstream target of xCT (Figure S3A). Taken together, these data demonstrate the central role of *OSGIN1* in SASP-induced PEL apoptosis, which involves modulation of a variety of host physiologic factors.

We previously reported some other AIDS-related lymphoma cell-lines such as Burkitt's lymphoma BL-41 and BJAB (both are KSHV^neg^/EBV^neg^) with highly expressed xCT, and SASP treatment induced significant apoptosis for BL-41 [5]. Here we found that silencing of *OSGIN1* by siRNA significantly reduced cell apoptosis induced by SASP (0.5 mM) in BL-41 cells (Figure S4). However, simply silencing of *OSGIN1* did not induce apoptosis for primary human CD19^+^ B cells isolated from peripheral blood of healthy donor (Figure S5).

### Targeting XRCC5 impairs DNA-damage repair abilities of tumor cells and promotes low dose of SASP-induced PEL apoptosis

**Figure 6:**
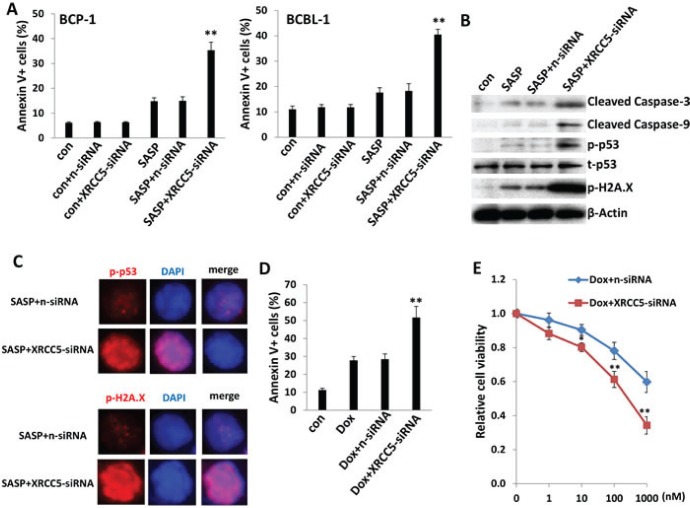
Targeting XRCC5 impairs DNA-damage repair abilities of PEL cells and promotes chemicals-induced apoptosis (**A**) BCP-1 and BCBL-1 were transfected with either negative control siRNA (n-siRNA) or *XRCC5*-siRNA for 48 h, then some cells incubated with 0.1mM of SASP for additional 24 h and cell apoptosis was assessed using Annexin V-PI staining and flow cytometry analysis. (**B**-**C**) Protein expression in BCBL-1 was measured by immuoblots and immunofluorescence, respectively. (**D**-**E**) BCBL-1 were transfected as (A), then incubated with 100 nM of doxorubicin (Dox) or indicated concentrations for additional 48 h, then cell apoptosis and death were measured by flow cytometry and MTT assay, respectively. Error bars represent the S.E.M. for 3 independent experiments. * = *p* < 0.05, ** = *p* < 0.01 (*vs* SASP+n-siRNA group or Dox+n-siRNA).

We were also interested in *XRCC5* (X-ray repair cross-complementing protein 5, also known as Ku80), one of downregulated genes in SASP-treated KSHV^+^ PEL cells, to determine its role in SASP-induced cell apoptosis. Ku80 is a tightly associated heterodimer of ~70 kDa and ~80 kDa subunits (Ku70 and Ku80) that, together with the ~470 kDa catalytic subunit, DNA-PKcs, form the DNA-dependent protein kinase involved in repairing DNA double-strand breaks (DSBs) caused by a variety of stress factors [31]. The Ku80-dependent repair process, called nonhomologous end joining (NHEJ), appears to be the main DNA DSB repair mechanism in mammalian cells [31, 32]. Interestingly, Ku80-knockout mice are small, and their cells fail to proliferate in culture and show signs of premature senescence [33, 34]. Here we found, for the first time, that silence of xCT by RNAi significantly downregulates *XRCC5* (Ku80) transcripts in BCBL-1 cells, indicating that *XRCC5* is also a downstream gene target of xCT (Figure S3B). Interestingly, direct siRNA silencing of *XRCC5* enhanced low-dose SASP (0.1mM)-induced PEL apoptosis, potentially due to impaired DNA-damage repair machinery in tumor cells (Figures 6A and S6). Immunoblot and immunofluorescence data further confirmed that silencing of *XRCC5* in low dose SASP-treated BCBL-1 cells increased the levels of cleaved Caspase 3 and 9 as well as phosphorylated p53 (Ser15) and phosphorylated Histone H2A.X (Ser139), the two markers for DNA-damage [35] (Figure 6B and 6C). Moreover, we found that silencing of *XRCC5* also enhanced the induction of apoptosis and programmed cell death by low dose concentrations of other DNA-damage reagents such as Doxorubicin (100 nM) induced PEL apoptosis (Figure 6D and 6E). Together, these data provide solid evidence that DNA-damage may represent another mechanism of SASP-induced PEL apoptosis/cell death, which is potentially through XRCC5. In addition, direct siRNA silencing of *XRCC5* also enhanced low-dose SASP (0.1mM)-induced apoptosis for Burkitt's lymphoma BL-41 cells (Figure S7), while targeting *XRCC5* induced no apoptosis for primary human CD19^+^ B cells isolated from peripheral blood of healthy donor (Figure S5).

## DISCUSSION

Recent studies have demonstrated that KSHV contributes to PEL survival and proliferation in part by downregulating or inactivating specific tumor suppressor genes. For example, KSHV-encoded latency-associated nuclear antigen (LANA) can directly interact with and inactivate the tumor suppressor functions of p53 and p73, thus promoting tumor cell survival [36, 37]. In addition, one of the KSHV microRNAs, miR-K12-1, directly targets and represses expression of p21, a well-known cyclin-dependent kinase (CDK) inhibitor, to promote PEL cell growth [38]. Furthermore, the amino acid transporter, xCT, which is highly expressed on the surface of KSHV^+^ PEL cells, also supports PEL growth not only through its amino-acid transport function but also through regulation of downstream cell survival effectors [5]. This is supported by our previous data demonstrating that the xCT selective inhibitor, SASP, blocks PEL tumor progression in an immune-deficient xenograft model [5], suggesting that the infectious process of KSHV is directly linked to post-entry mechanisms involved in virus-associated lymphomagenesis, but the mechanisms by which xCT orchestrates this link are not fully defined.

In the current study, we used microarray analysis to interrogate the transcriptional profile of SASP-treated KSHV^+^ PEL cell lines, and identified a number of genes whose expression was altered in a unique and global manner. Enrichment analysis indicated that targeting xCT in this manner resulted in upregulated expression of a class of genes that may function to promote PEL cell survival in part by preventing apoptosis and/or programmed cell death, implying that in addition to its natural function as amino acid transporter, xCT also acts as a global regulator of down-stream effector proteins involved in tumor cell survival.

First we discovered that *OSGIN1*, a tumor suppressor gene that is upregulated in SASP-treated PEL cells, plays a role in SASP-induced PEL apoptosis through regulation of Akt signaling, GSH synthesis and ROS production. Interestingly, the OSGIN1 homolog, bone marrow stromal cell (BMSC)-derived growth inhibitor (BDGI), has been shown to induce cell cycle arrest in S phase and subsequent apoptosis of MCF-7 breast cancer cells, which potentially occurs through upregulation of p27^Kip1^ and downregulation of cyclin A, Bcl-2, and Bcl-xL [39]. Therefore, our data are consistent with an emerging theme with respect to xCT-mediated tumor cell survival, and sets the stage for derivative studies aimed at determining whether targeting the xCT/OSGIN1 axis will also impact proteins involved in the PEL cell cycle.

Doxorubicin, a DNA-damage reagent, is one of the first-line chemotherapy drugs for PEL treatment [3]. However, our previous studies have demonstrated that some KSHV^+^ PEL cell-lines (e.g. BCP-1 and BCBL-1) display multidrug chemoresistance to a number of chemotherapeutic drugs, including Doxorubicin [40]. Here we found that siRNA “knock-down” of *XRCC5*, one of the downstream genes regulated by xCT, impaired the DNA-damage repair machinery and sensitized BCBL-1 to low dose Doxorubicin-induced cell apoptosis/cell death. This result is consistent with a previous study in which Huang *et al.* demonstrated a link between the level of xCT expression in a panel of cancer cell lines with the potency of 1,400 candidate anticancer drugs, with 39 positive correlations, and 296 negative correlations [41]. Therefore, we have reason to believe that targeting xCT/XRCC5 represents a promising “combination” strategy for enhancing the efficacy of chemotherapeutic drugs while reducing systemic cytotoxicity.

Considered in a broader context, our data supports evaluation of xCT targeting as a means to attenuate survival and/or growth of other non-KSHV-associated lymphomas as well. For instance, xCT is expressed on some Burkitt's lymphoma cell-lines such as BL-41 and BJAB (both KSHV^neg^/EBV^neg^), AKATA (KSHV^neg^/EBV^+^) and on some diffuse large cell lymphoma (DLCL) cell-lines such as CRL2631 (KSHV^neg^/EBV^neg^), and inhibition of xCT by SASP also induced significant apoptosis in BL-41 lymphoma cells expressing high levels of xCT [5]. Therefore, it will be interesting to explore the global gene profile altered by targeting xCT in other AIDS-related lymphoma cells as well. As mentioned above, SASP treatment induced a much higher number of uniquely altered genes in the dually infected BC-1 (KSHV^+^/EBV^+^) cells than in BCBL-1 and BCP-1 (both of which are KSHV^+^/EBV^neg^), suggesting that complex interactions between these co-existent oncogenic herpesviruses may influence the outcome of SASP treatment. In this respect subtractive microarray analysis of differential gene expression cell lines latently infected with one or both viruses may reveal important themes related to their strategies for persistence and induction of associated malignancies.

## MATERIALS AND METHODS

### Cell culture and reagents

The PEL cell-line BCBL-1 (KSHV^+^/EBV^neg^) and a Burkitt's lymphoma cell line BL-41 (KSHV^neg^/EBV^neg^) was kindly provided by Dr. Dean Kedes (University of Virginia) and maintained in RPMI 1640 medium (Gibco) with supplements as described previously [42]. The other PEL cell-lines BC-1 (KSHV^+^/EBV^+^) and BCP-1 (KSHV^+^/EBV^neg^) were purchased from American Type Culture Collection (ATCC) and maintained in complete RPMI 1640 medium (ATCC) supplemented with 20% FBS. All cells were cultured at 37°C in 5% CO_2_. All experiments were carried out using cells harvested at low (< 20) passages. Sulfasalazine (SASP) and Doxorubicin were purchased from Sigma.

### Microarray

Microarray analysis was performed and analyzed at the Stanley S. Scott Cancer Center's Translational Genomics Core at LSUHSC. BC-1, BCP-1 and BCBL-1 cells were treated with vehicle or the xCT selective inhibitor SASP (0.5 mM) for 48 h, respectively. Total RNA was isolated using Qiagen RNeasy kit (Qiagen), and 500 ng of total RNA was used to synthesize dscDNA. Biotin-labeled RNA was generated using the TargetAmp-Nano Labeling Kit for Illumina Expression BeadChip (Epicentre), according to the manufacturers’ instructions, and hybridized to the HumanHT-12 v4 Expression BeadChip (Illumina) which contains more than 47,000 probes derived from the NCBI RefSeq Release 38 and other sources, at 58°C for 16 h. The chip was washed, stained with streptavadin-Cy3, and scanned with the Illumina BeadStation 500 and BeadScan. Using the Illumina's GenomeStudio software, we normalized the signals using the “cubic spline algorithm” that assumes that the distribution of the transcript abundance is similar in all samples, according to the method proposed by Workman *et al.* [43]. The background signal was removed using the “detection *p*-value algorithm” to remove targets with signal intensities equal or lower than that of irrelevant probes (with no known targets in the human genome but thermodynamically similar to the relevant probes). The microarray experiments were performed twice for each group and the average values were used for analysis. Common, similar, and unique sets of genes and enrichment analysis were performed using the MetaCore Software (Thompson Reuters) as previously reported [23]. The microarray original data have been submitted to Gene Expression Omnibus (GEO) database (Accession number: GSE65418).

### Isolation of circulating human B cells

Human peripheral blood mononuclear cells (PBMC) were isolated from whole blood from the healthy donor following Ficoll gradient separation. PBMC were washed and resuspended in 500 μL total volume, including 440 μL buffer composed of 2% FBS and 1 mM EDTA in 1X PBS (EasySep buffer, STEMCELL Technologies), 30 μL Fc-receptor blocker (eBiosciences), and 30 μL of a PE-conjugated anti-CD19 monoclonal antibody (BD-Pharmagen), for incubation at RT for 20 minutes. 100 μL EasySep PE selection cocktail (STEMCELL Technologies) was added for an additional 15 minutes, and 2.5 mL of additional buffer was then added prior to magnetic column separation of CD19^+^ cells. Following column separation, supernatants were discarded and cells resuspended in fresh 2.5 mL buffer for each of two additional column separation steps. Thereafter, cells were resuspended in complete RPMI 1640 medium supplemented with 20% FBS for further experiments, or in 1X PBS for flow cytometry to determine the purity of selection. 92-95% pure populations of CD19^+^ cells were recovered (data not shown).

### Cell viability assays

Cell viability was assessed using MTT assays for assessment of proliferative capacity, and flow cytometry was used for quantitative assessment of apoptosis. Standard MTT assays were performed as described previously [5]. For flow cytometry, the FITC-Annexin V/propidium iodide (PI) Apoptosis Detection Kit I (BD Pharmingen) was used according to the manufacturer's instructions.

### Immunoblotting

Cells were lysed in buffer containing 20 mM Tris (pH 7.5), 150 mM NaCl, 1% NP40, 1 mM EDTA, 5 mM NaF and 5 mM Na_3_VO_4_. Total cell lysates (30 μg) were resolved by 10% SDS–PAGE, transferred to nitrocellulose membranes, and immunoblotted using 100-200 μg/mL antibodies to cleaved-caspase 3/9, p-Akt, p-P70S6, p-S6, p-H2A.X, p-p53/t-p53, and XIAP (all purchased from Cell Signaling, Inc., Danvers, MA). For loading controls, blots were incubated with antibodies detecting β-Actin (Sigma). Immunoreactive bands were developed using an enhanced chemiluminescence reaction (Perkin-Elmer) and visualized by autoradiography.

### Immunofluorescence assays (IFA)

Cells were incubated in 1:1 methanol-acetone at −20°C for fixation and permeabilization, then with a blocking reagent (10% normal goat serum, 3% bovine serum albumin, and 1% glycine) for an additional 30 minutes. Cells were then incubated for 1 h at 25°C with 1:400 dilution of a mouse anti-p-p53 antibody or a rabbit anti-p-H2A.X antibody (Cell Signaling) followed by 1:200 dilution of a goat anti-mouse or goat anti-rabbit secondary antibody conjugated with Texas Red (Invitrogen), respectively. For identification of nuclei, cells were subsequently counterstained with 0.5 μg/mL 4′,6-diamidino-2-phenylindole (DAPI; Sigma) in 180 mM Tris-HCl (pH 7.5). Cells were washed once in 180 mM Tris-HCl for 15 minutes and prepared for visualization using a Leica TCPS SP5 AOBS confocal microscope.

### RNA interference

For RNA interference assays, ON-TARGET plus SMART pool siRNA for *xCT*, *OSGIN1* or *XRCC5* (Dharmacon), or negative control siRNA, were delivered using the DharmaFECT transfection reagent according to the manufacturer's instructions.

### qRT-PCR

Total RNA was isolated using the RNeasy Mini kit according to the manufacturer's instructions (QIAGEN). cDNA was synthesized from equivalent total RNA using SuperScript III First-Strand Synthesis SuperMix Kit (Invitrogen) according to the manufacturer's procedures. Primers used for amplification of target genes are displayed in Table S1. Amplification was carried out using an iCycler IQ Real-Time PCR Detection System, and cycle threshold (Ct) values were tabulated in duplicate for each gene of interest in each experiment. “No template” (water) controls were used to ensure minimal background contamination. Using mean Ct values tabulated for each gene, and paired Ct values for β-actin as an internal control, fold changes for experimental groups relative to assigned controls were calculated using automated iQ5 2.0 software (Bio-rad).

### ROS measurement

PEL cells were loaded with 10 μM of the ROS dye c-H2DCFDA (Invitrogen) for 30 min at 37°C in Hanks’ Balanced Salt Solution (HBSS) containing calcium and magnesium (HBSS/Ca/Mg). Cells were then washed once with HBSS/Ca/Mg to remove dye, resuspended in HBSS/Ca/Mg and subjected to flow cytometry analysis as previously described [5].

### NADPH oxidase activities assays

The chemiluminescence-based NADPH oxidase activity assays were performed as described previously [5]. After drug-treatment, cells were centrifuged at 500 g for 10 min at 4°C. The cell pellet was resuspended in 35 μL ice-cold lysis buffer and kept on ice for 20 min. To a final 200 μL of HBSS/Ca/Mg buffer containing NADPH (1 μM, Sigma) and lucigenin (20 μM, Sigma), 5 μL of cell lysates was added to initiate the reaction for 5 min at 37°C. Chemiluminescence was measured immediately using a Synergy HT microplate reader (BioTek Instruments).

### Intracellular GSH measurement

The intracellular GSH levels in PEL cells were quantified using the GSH-Glo™ Glutathione Assay Kit (Promega), according to the manufacturer's instructions.

### Statistical analyses

Significance for differences between experimental and control groups was determined using the two-tailed Student's t-test (Excel 8.0).

## SUPPLEMENTARY MATERIAL FIGURES AND TABLE


