# Supplementary material for: Genomic analysis of xCT-mediated regulatory network: identification of novel targets against AIDS-associated lymphoma
Source: Oncotarget. 2015 Mar 30;6(14):12710–22. doi: 10.18632/oncotarget.3710 (PMC4494968; doi:10.18632/oncotarget.3710)
Supplement: Supplementary file 1 [file oncotarget-06-12710-s001.pdf]

Genomic analysis of xCT-mediated regulatory network: identification of novel targets against AIDS-associated lymphoma

Supplementary Material

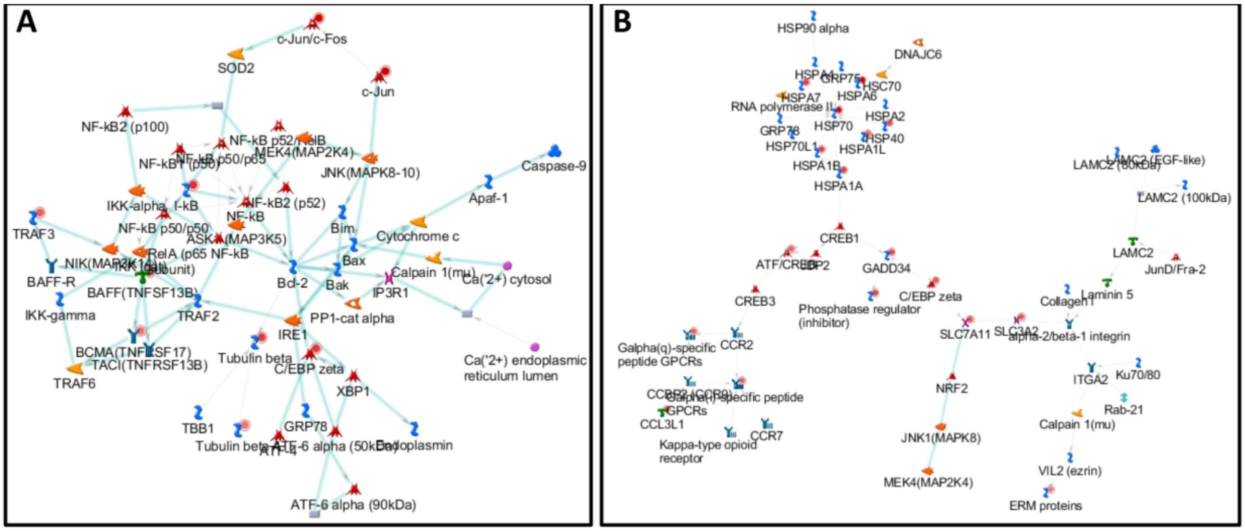

Supplemental Figure 1: The top 2 scored (by the number of pathways) AN networks from 'common' gene set. Thick cyan lines indicate the fragments of canonical pathways. Upregulated genes are marked with red circles; downregulated with blue circles. The 'checkerboard' color indicates mixed expression for the gene between files or between multiple tags for the same gene. Data was produced by the Metacore Software (Thompson Reuters).

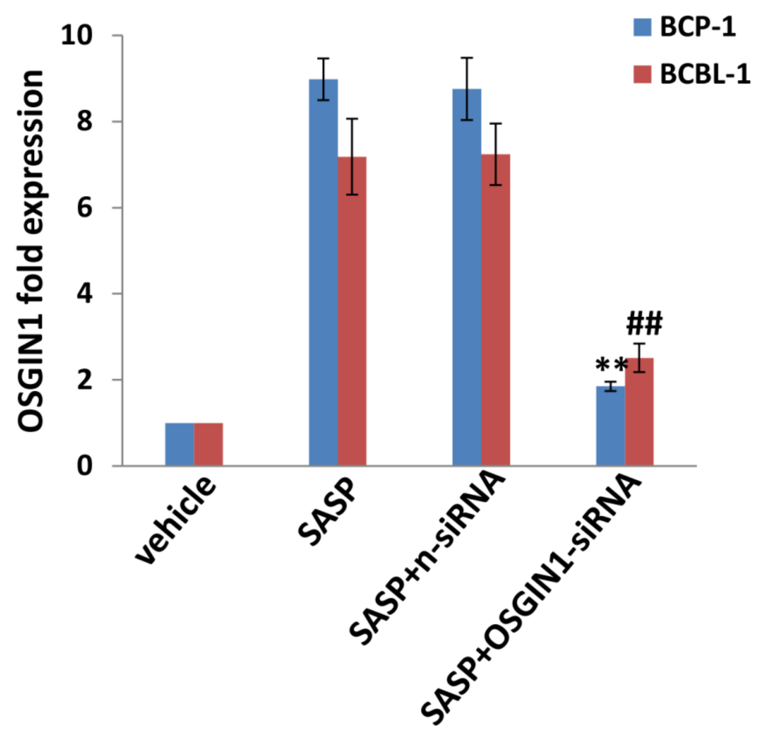

Supplemental Figure 2: Successful knock-down OSGIN1 by RNAi from KSHV-infected PEL cells. BCP-1 and BCBL-1 were transfected with either negative control siRNA (n-siRNA) or *OSGIN1*-siRNA for 48 h, then incubated with 0.5mM of SASP for additional 24 h and gene transcripts were quantified by qRT-PCR. Error bars represent the S.E.M. for 3 independent experiments, **/## = $p<0.01$ (*vs* SASP+n-siRNA group).

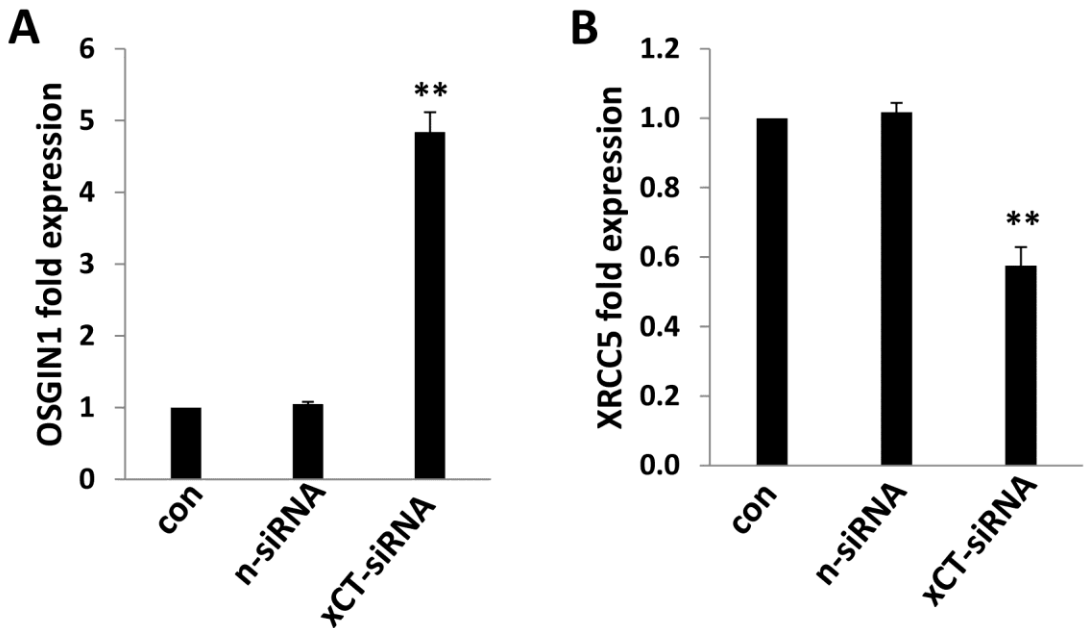

Supplemental Figure 3: xCT regulates OSGIN1 and XRCC5 transcription within KSHV-infected PEL cells. BCBL-1 were transfected with either negative control siRNA (n-siRNA) or *xCT*-siRNA for 48 h, then gene transcripts were quantified by qRT-PCR. Error bars represent the S.E.M. for 3 independent experiments, **= p<0.01 (*vs* n-siRNA group).

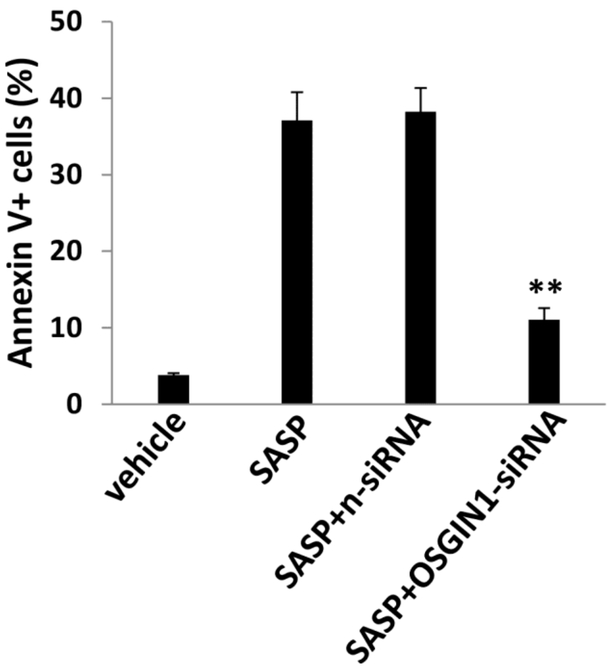

Supplemental Figure 4: SASP induces Burkitt's lymphoma cell apoptosis potentially through OSGIN1. Burkitt's lymphoma BL-41 cells were transfected with either negative control siRNA (n-siRNA) or *OSGIN1*-siRNA for 48 h, then incubated with 0.5mM of SASP for additional 24 h and cell apoptosis was assessed using Annexin V-PI staining and flow cytometry analysis. Error bars represent the S.E.M. for 3 independent experiments, **= p<0.01 (*vs* SASP+n-siRNA group).

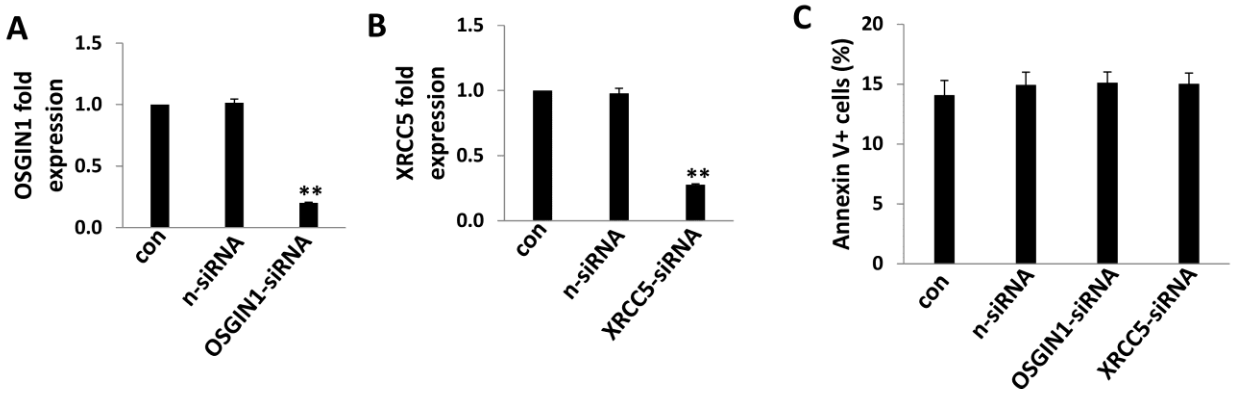

Supplemental Figure 5: Direct silencing of *OSGIN1* or *XRCC5* induces no apoptosis for circulating human B cells. Human $CD19^+$ cells were isolated from peripheral blood collected from healthy donor as described in Methods. Cells were then transfected with either negative control siRNA (n-siRNA), *OSGIN1*-siRNA or *XRCC5*-siRNA for 48 h, and gene expression (**A-B**) or cell apoptosis (**C**) were measured as described above. Error bars represent the S.E.M. for 3 independent experiments, **= p<0.01 (*vs* n-siRNA group).

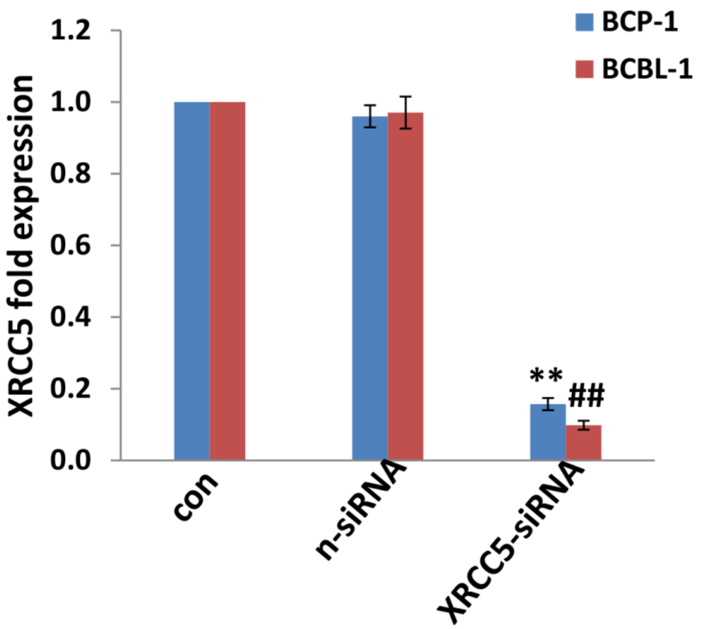

Supplemental Figure 6: Successful knock-down XRCC5 by RNAi from KSHV-infected PEL cells. BCP-1 and BCBL-1 were transfected with either negative control siRNA (n-siRNA) or *XRCC5*-siRNA for 48 h, then gene transcripts were quantified by qRT-PCR. Error bars represent the S.E.M. for 3 independent experiments, **/## = p<0.01 (*vs* n-siRNA group).

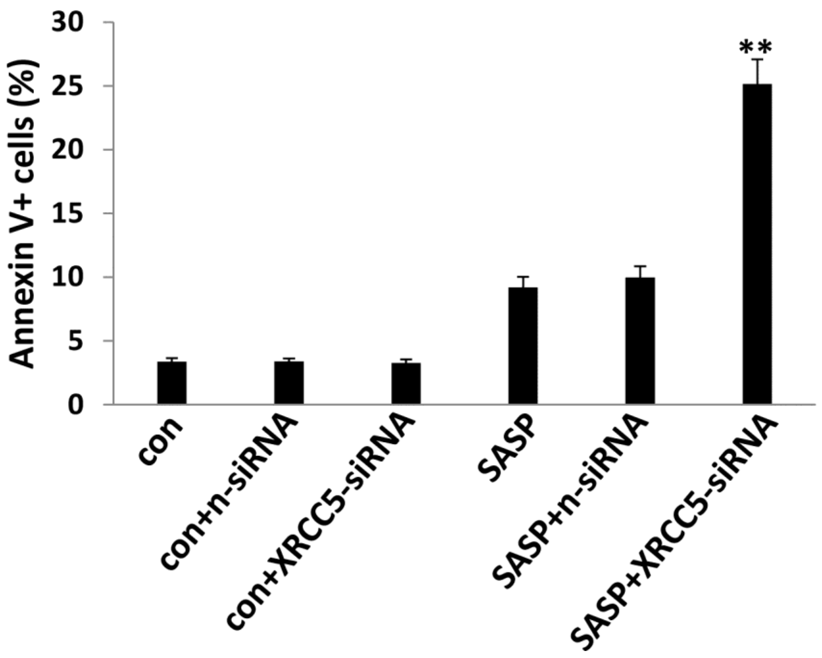

Supplemental Figure 7: Targeting XRCC5 promotes low dose of SASP induced BL-41 apoptosis. BL-41 were transfected with either negative control siRNA (n-siRNA) or *XRCC5*-siRNA for 48 h, then some cells incubated with 0.1mM of SASP for additional 24 h and cell apoptosis was assessed using Annexin V-PI staining and flow cytometry analysis. Error bars represent the S.E.M. for 3 independent experiments, **= $p<0.01$ (*vs* SASP+n-siRNA group).

Supplemental Table 1: Primer sequences for qRT-PCR in this study.

Gene	Sequences (5' ⟶ 3')
ASZ1	*sense GTATGCTGCTAGTGTTGC*
	antisense ATTCTCATCCTGGGTATT
ARL4C	*sense TCGTCATGTTGGGCTTGG*
	antisense GCTTGATCTTCTCGGTGTTG
NREP	*sense TTATTACCCAGAACTCTTTGTC*
	antisense CAGCGTTTGTCTCATCGT
LGALS13	*sense TCTTTACCCGTGCCATAC*
	antisense TCTCCTCCAACATCCATA
PPIA	*sense ATTATTCCAGGGTTTATGTG*
	antisense AACTGGGAACCATTTGTG
XRCC5	*sense CTGGAGGACATTGAAAGC*
	antisense AGAAGAATTGCAGGGAGA
OSGIN1	*sense CCCTGTCCTCATCATTGGC*
	antisense GGCTGCGGTAACCCTCATAG
HSPA6	*sense GGGACAAATGTGAGAAAGTGC*
	antisense GTAGGTGGTGAAAGTCTGGGT
DHRS2	*sense CCAAGGACATCCGGGTAA*
	antisense CAGAGCCGAGTGGAGTAGC
PPP1R15A	*sense AACCTCTACTTCTGCCTTGTC*
	antisense CCTTTCCCAGTTTCTTTGTG
HSPA1A	*sense GAACAAGCGAGCCGTGAG*
	antisense AGGGTGCTTCGGAACAGG
β-actin	*sense GGAAATCGTGCGTGACATT*
	antisense GACTCGTCATACTCCTGCTTG
